# The Medical Session

**Published:** 1897-10-09

**Authors:** 


### Medical Progress and Hospital Clinics.

[*The Editor will be glad to receive offers of co-operation and contributions from members of the profession. All letters should be addressed to* The Editor, at the Office, 28 & 29, Southampton Street, Strand, London, W.C.]

### The Medical Session.

The 1st of October is an anniversary which arouses the medical profession of Great Britain to renewed activity, and which recalls its members to labour after any refreshment which they may have been able to obtain from holidays. The hospital schools reopen their doors, both to welcome old pupils and to provide for the reception and instruction of new ones; and the authorities of these schools vie with each other in the attractions which they offer. At most of them the old custom of an introductory lecture is adhered to, and either some distinguished member of the staff, or some still more distinguished stranger, is required to descant for an hour upon the attractions of a medical career, upon the opportunities which it affords, and upon the methods by which these opportunities may most effectually be utilised. On this last branch of the subject it is highly probable that each student, if of average capacity and industry, may safely be left to be a law unto himself. There is an old story of a Scottish laird who brought home the intelligence that he had invited a large party to dine and sleep, and whose wife tearfully asserted that the house did not contain sufficient beds to meet the requirements of the expected visitors. "Dinna fash yoursel', Maggie," replied the laird, "gin you gie whuskey eneuch they'll just find beds for theirsells." Very much the same thing might be said for the medical students of to-day, if for the potent wine of North Britain we might substitute the still more potent stimulant of knowledge. If a medical man be well taught, and if he has received his teaching with a determination to turn it to profitable account, so as to qualify himself to do his duty to his patients, be they rich or poor, great or small, he will be quite certain to find himself a bed somewhere. The difficulties of a medical career come chiefly to those who regard the calling as a trade, and who are more solicitous about the remuneration they will receive than about the cures which they may effect, for the profession affords only a mediocre prospect to any who are unable to realise the difference between skill and merchandise.

Among the introductory addresses of the present year, few have been more noteworthy than that in which Mr. Jessop, at Leeds, described the provision which is made for medical education in Moscow, and spoke of it as affording an example to Europe. The school buildings have been erected at a cost of half a million sterling, and they have an endowment of £43,000 a year, derived from grants made partly by the Imperial Government and partly by the municipality of the city. Mr. Jessop did not state on what conditions students are admitted, or what fees they are called upon to pay; but it may be remembered that the population of the city is about a million. The medical schools of London are private undertakings, which do not publish their accounts; but it may be surmised that their combined incomes, from the fees of students, are about equal to the Moscow endowment, and it is certain that, out of these payments, the whole of their expenses must be defrayed before the teachers attached to them can receive a farthing. The tendency is clearly to cripple teaching, although its effects are greatly modified by the rivalry between schools, and by the devotion to science which leads many teachers to work for very slender payment, under the influence of enthusiasm for the diffusion of truth and for the advancement of science. Powerful as these influences are, they are not omnipotent, and such a school as that of Moscow cannot but threaten the supremacy of the United Kingdom as the home and centre of medical science. It would be deplorable if the nation which has given to the world railways, electric telegraphs, steam navigation, and cheap postage, and which, in the domain of medicine, has given the knowledge of the circulation, of spinal function, of reflex action, of asepsis in surgery, and of the safe removal of ovarian cysts, should be impeded in its onward course by financial difficulties which a community less advanced in civilisation has found it possible to overcome. It may be that Russia is less encumbered than England by a class of persons who constitute a serious impediment alike to medical teaching and to medical progress—a class which has been known and studied from time immemorial, and of which a representative, almost certainly an "anti" something or other, was described by King Solomon as being "wiser in his own conceit than seven men who could render a reason." We are not without hope that, if the catastrophe of the Gloucester small-pox epidemic, or the catastrophe of the Maidstone typhoid epidemic, had happened in Russia, it might have been found possible to hang somebody, and thus to inflict upon the murderer of a large number of fellow creatures the punishment which is thought appropriate for the murderer of one. Before anything like this can be done with complete public approval among ourselves, it will be necessary for the facts of medical science to sink more deeply into the mind of the man in the street than they have done hitherto.

